# COVID-19 and liver cancer: lost patients and larger tumours

**DOI:** 10.1136/bmjgast-2021-000794

**Published:** 2022-04-21

**Authors:** Daniel Geh, Robyn Watson, Gourab Sen, Jeremy J French, John Hammond, Paul Turner, Tim Hoare, Kirsty Anderson, Michael McNeil, Stuart McPherson, Steven Masson, Jessica Dyson, Mhairi Donnelly, Louise MacDougal, Preya Patel, Mark Hudson, Quentin M Anstee, Steven White, Stuart Robinson, Sanjay Pandanaboyana, Lucy Walker, Misti McCain, Yvonne Bury, Shreya Raman, Alastair Burt, Daniel Parkinson, Beate Haugk, Antony Darne, Nick Wadd, Syed Asghar, Lavanya Mariappan, Jane Margetts, Benjamin Stenberg, John Scott, Peter Littler, Derek M Manas, Helen L Reeves

**Affiliations:** 1 Translational and Clinical Research Institute, Faculty of Medical Sciences, Newcastle University, Newcastle upon Tyne, UK; 2 Hepatopancreatobiliary Multidisciplinary Team, Newcastle upon Tyne Hospitals NHS Foundation Trust, Newcastle upon Tyne, UK; 3 Department of Surgery, Newcastle upon Tyne Hospitals NHS Foundation Trust, Newcastle upon Tyne, UK; 4 Department of Radiology, Newcastle upon Tyne Hospitals NHS Foundation Trust, Newcastle upon Tyne, UK; 5 The Liver Unit, Department of Medicine, Newcastle upon Tyne Hospitals NHS Foundation Trust, Newcastle upon Tyne, UK; 6 Discovery Medicine, GlaxoSmithKline Plc, Brentford, UK; 7 Department of Cellular Pathology, Newcastle upon Tyne Hospitals NHS Foundation Trust, Newcastle upon Tyne, UK; 8 Department of Oncology, South Tees Hospitals NHS Foundation Trust, Middlesbrough, UK; 9 Department of Oncology, North Cumbria Integrated Care NHS Foundation Trust, Carlisle, UK; 10 Northern Centre for Cancer Care, Newcastle upon Tyne Hospitals NHS Foundation Trust, Newcastle upon Tyne, UK

**Keywords:** HEPATOCELLULAR CARCINOMA, CHOLANGIOCARCINOMA, COVID-19, SCREENING, SURVEILLANCE

## Abstract

**Background:**

Northern England has been experiencing a persistent rise in the number of primary liver cancers, largely driven by an increasing incidence of hepatocellular carcinoma (HCC) secondary to alcohol-related liver disease and non-alcoholic fatty liver disease. Here we review the effect of the COVID-19 pandemic on primary liver cancer services and patients in our region.

**Objective:**

To assess the impact of the COVID-19 pandemic on patients with newly diagnosed liver cancer in our region.

**Design:**

We prospectively audited our service for the first year of the pandemic (March 2020–February 2021), comparing mode of presentation, disease stage, treatments and outcomes to a retrospective observational consecutive cohort immediately prepandemic (March 2019–February 2020).

**Results:**

We observed a marked decrease in HCC referrals compared with previous years, falling from 190 confirmed new cases to 120 (37%). Symptomatic became the the most common mode of presentation, with fewer tumours detected by surveillance or incidentally (% surveillance/incidental/symptomatic; 34/42/24 prepandemic vs 27/33/40 in the pandemic, p=0.013). HCC tumour size was larger in the pandemic year (60±4.6 mm vs 48±2.6 mm, p=0.017), with a higher incidence of spontaneous tumour haemorrhage. The number of new cases of intrahepatic cholangiocarcinoma (ICC) fell only slightly, with symptomatic presentation typical. Patients received treatment appropriate for their cancer stage, with waiting times shorter for patients with HCC and unchanged for patients with ICC. Survival was associated with stage both before and during the pandemic. 9% acquired COVID-19 infection.

**Conclusion:**

The pandemic-associated reduction in referred patients in our region was attributed to the disruption of routine healthcare. For those referred, treatments and survival were appropriate for their stage at presentation. Non-referred or missing patients are expected to present with more advanced disease, with poorer outcomes. While protective measures are necessary during the pandemic, we recommend routine healthcare services continue, with patients encouraged to engage.

**Summary box**

**What is already known about this subject?**

- Deaths from hepatocellular carcinoma (HCC) have been rising year on year in the North East and Cumbria, attributed to increases in non-alcoholic fatty liver disease and alcohol-related liver disease.
- Detection as part of routine clinical care, before symptoms, can save lives.
- The COVID-19 pandemic has caused an unprecedented burden on healthcare resources, with chronic disease management and routine health checks disrupted.

**What are the new findings?**

- The COVID-19 pandemic has resulted in a 37% reduction in the number of new HCC cases detected in our region. Fewer have been detected by surveillance or as part of routine care, with an increase in symptomatic, larger tumours.
- Presenting patients have received a full range of curative and palliative therapies during the pandemic.

**How might it impact on clinical practice in the foreseeable future?**

- Our absent patients are missing treatment opportunities. We anticipate their symptomatic presentation with larger tumours in the coming months and years.
- As the pandemic evolves, we recommend that routine primary and secondary care services are supported and patients encouraged to engage.
  - Patients should follow current government guidance regarding vaccination, personal protective equipment and social distancing.
  - Delays in surveillance should be not be longer than 3 months if possible, in all patients who would benefit from cancer treatments.
  - Guided by multidisciplinary discussion, all liver cancer treatments should be considered for eligible patients, as per standard guidelines.

## Introduction

Since the start of the COVID-19 pandemic there have been over 290 million confirmed infections and 5 million deaths reported worldwide.^1^ Because of the unprecedented burden on healthcare resources, many healthcare activities such as chronic disease management, cancer screening and cancer treatments have been cancelled or delayed. Consequently, referrals of suspected new cancers have reduced, with increases in cancer-related deaths predicted.^2^ The full impact of the COVID-19 pandemic on patients with primary liver cancer (PLC) has yet to be determined, although European data reported a disruption to hepatocellular carcinoma (HCC) services, a reduction in incident cases and an impact on management during the first wave of the pandemic (February 2020 to May 2020).^3–6^

The incidence of PLC has been increasing in the UK and globally.^7 8^ In Northern England increases in deaths of patients with HCC in recent years have been attributed to the rising prevalence of obesity-associated non-alcoholic fatty liver disease (NAFLD), alongside alcohol-related liver disease (ARLD).^9^ While the UK experienced its first and second waves of the COVID-19 pandemic between March 2020–June 2020 and October 2020–April 2021, respectively, it was the second wave that was particularly sustained in the North of England.^10^ As the pandemic continues, we have evaluated the impact of COVID-19 in our region during its first year (first wave and second wave until the end of February 2021), raising key learning points and making recommendations for future service provision.

## Methods

In North East England and Cumbria (population ~3.5 million), all suspected patients with PLC are referred to the Newcastle upon Tyne NHS Foundation Trust (NUTH) hepatopancreatobiliary multidisciplinary team (MDT). This study was approved as an audit by NUTH Research and Development Department (Audits 10170; 10148; Caldicott Register 7606), prospectively considering all patients referred in the first 12 months of the pandemic (March 2020–February 2021), comparing to a retrospective observational cohort of consecutive patients presenting in the 12 months immediately preceding it (March 2019–February 2020). All new cases with a diagnosis of HCC or intrahepatic cholangiocarcinoma (ICC) confirmed radiologically or histologically, following international guidelines,^11 12^ were included. Data on diagnosis, mode of presentation, adherence to surveillance, underlying disease aetiology, disease stage, treatment and COVID-19 infection were recorded. The date of reference for comparative analyses was the date of first discussion by our MDT, with this taken as the date of diagnosis. Survival was recorded until the 29^th^ November 2021, obtained from electronic healthcare records. Mode of presentation was categorised as ‘surveillance’, ‘incidental’ or ‘symptomatic’. Incidental presentations included asymptomatic cancers detected in the primary or secondary setting as part of routine care or the investigation of an unassociated condition. For those undergoing surveillance, presentation was classed as incidental or symptomatic if cancers were detected outside a surveillance scan. Adherence to surveillance was classified as ‘consistent’ (6 monthly ultrasound (US)±alpha-fetoprotein (AFP) for the preceding year or since diagnosis of cirrhosis); ‘inconsistent’ (some US±AFP in the preceding year or since diagnosis of cirrhosis) or ‘missed’ (in a surveillance programme but completely missed US±AFP in the preceding year or since diagnosis of cirrhosis). The interval between surveillance scans was recorded both before and during the pandemic.

Statistical analyses employed IBM SPSS statistics software V.27. Differences between continuous datasets were assessed with unpaired t-tests and Mann-Whitney U tests for parametric and non-parametric datasets, respectively. Differences between categorical datasets were assessed using Pearson’s χ^2^ tests, with the Monte Carlo correction for datasets including counts less than 5. Survival analyses were by the Kaplan-Meier method, using a log-rank test.

## Results

### New HCC cases fell, with fewer detected by surveillance or incidentally discovered

**Figure 1:**
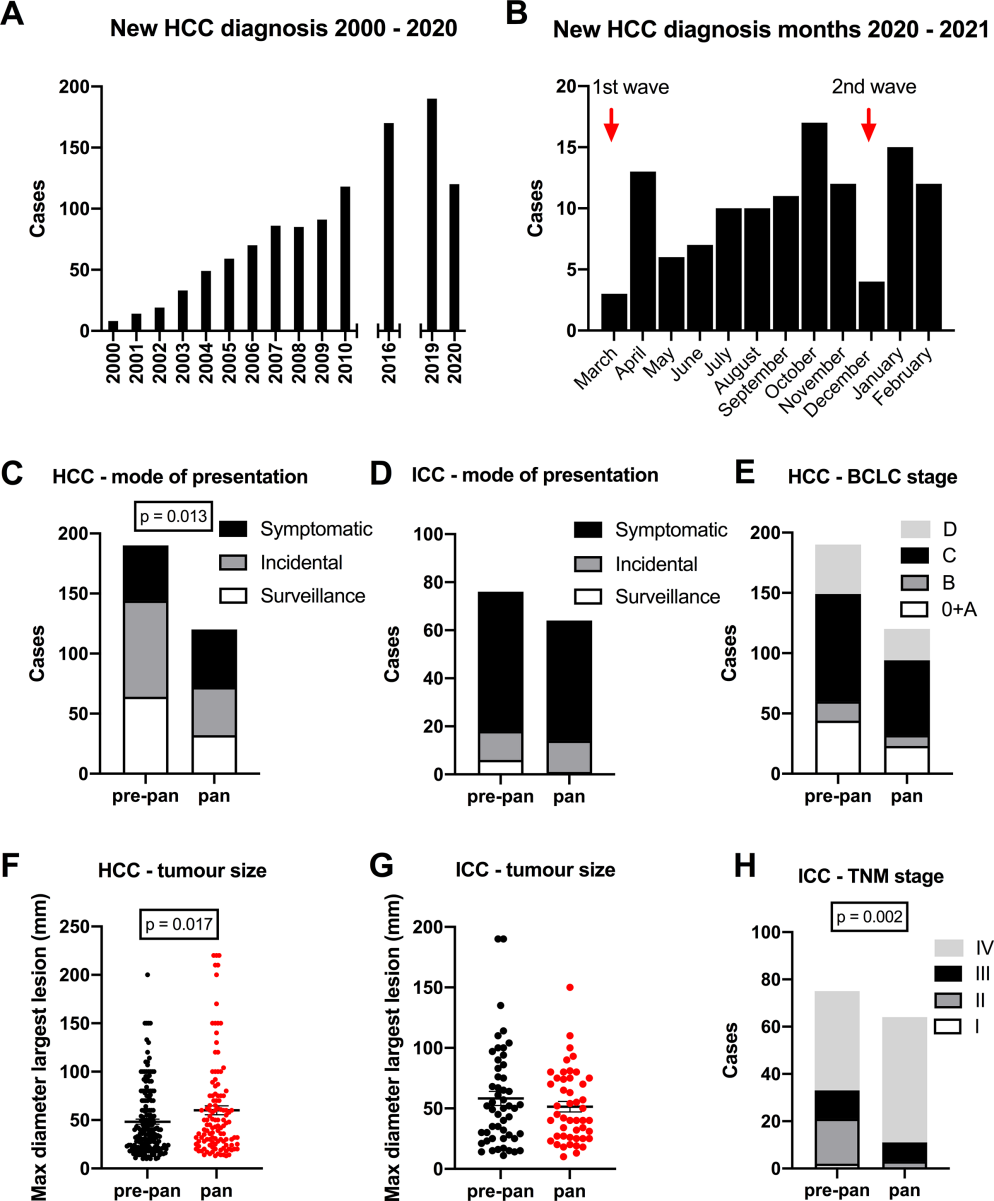
Primary liver cancers and the impact of COVID-19 on presentation and stage. The numbers of new hepatocellular carcinomas (HCCs) diagnosed in the North of England fell in the pandemic year compared with previous years (A), particularly at the peaks of the UK pandemic (B). Fewer cases were detected by surveillance or routine care in the pandemic (pan), compared with prepandemic (pre-pan) year, with more presenting symptomatically (C). Numbers of intrahepatic cholangiocarcinomas (ICCs) diagnosed in the pandemic year also fell, with the majority presenting symptomatically (D). Fewer Barcelona Clinic for Liver Cancer (BCLC) stage 0-B HCCs were detected in the pandemic year although this was not significantly different as a proportion of all cases referred (E). The mean HCC tumour size was significantly elevated in the pandemic year (F). During the pandemic year, the size of ICC tumours remained the similar (G) but significantly more ICCs were staged at an advanced tumour-node-metastases (TNM) stage (H).

During the pandemic year there were 120 new HCC diagnoses compared with 190 in the prepandemic year (figure 1A). This 37% decrease was in contrast to the rising numbers recorded in our region in previous years (figure 1A). Cases fell particularly in the months corresponding to the peaks of the first and second waves of the UK pandemic (figure 1B). When cases were separated according to aetiology, there were no significant differences overall (table 1, p=0.059), although the reduction was most apparent for patients with hepatitis C virus (HCV)-associated HCC (20 HCV-HCC cases in the prepandemic year vs 4 in the pandemic year). NAFLD was the the most common underlying cause in both years. In the pandemic year, the numbers of cases detected by surveillance or incidentally were halved, while symptomatic presentations were similar (% surveillance/incidental/symptomatic; 34/42/24 prepandemic vs 27/33/40 in the pandemic, Pearson’s χ^2^ p=0.013). Proportionally, symptomatic presentations rose from 24% to 40% (figure 1C and table 1).

**Table 1:** Clinical details of patients with HCC

		Year		P value
		Prepandemic (March 2019–February 2020)	Pandemic (March 2020–February 2021)	
Cases diagnosed		190	120	
Mode of presentation	Surveillance	64 (34%)	32 (27%)	p=0.013*
	Incidental	80 (42%)	40 (33%)	
	Symptomatic	46 (24%)	48 (40%)	
Underlying liver disease	Cirrhosis	134 (71%)	80 (67%)	p=0.474
	No cirrhosis	56 (29%)	40 (33%)	
Aetiology	ARLD	62 (32%)	33 (27%)	p=0.059
	NAFLD	69 (36%)	51 (42%)	
	HCV	20 (10%)	4 (3%)	
	HBV	3 (2%)	2 (2%)	
	HH	6 (5%)	9 (8%)	
	PBC/AIH	8 (4%)	3 (3%)	
	Other	0 (0%)	2 (2%)	
	No established CLD	22 (11%)	16 (13%)	
Age in years (median)		70.95±0.73 (72)	70.59±0.98 (71.5)	p=0.921
Gender	Male	146 (77%)	101 (84%)	p=0.119
	Female	44 (23%)	19 (16%)	
Performance status	0	56 (30%)	38 (32%)	p=0.530
	1	68 (36%)	32 (27%)	
	2	37 (20%)	30 (25%)	
	3	26 (13%)	17 (14%)	
	4	2 (1%)	2 (2%)	
Tumour diameter in mm (median)		48±2.6 (37)	60±4.6 (44)	p=0.017*
Tumour number		1.87±0.10	2.18±0.15	p=0.070
Portal vein thrombosis		44 (24%)	34 (29%)	p=0.371
Extrahepatic disease		26 (14%)	17 (14%)	p=0.940
Albumin (g/L) mean		38.0±0.49	38.2±0.60	p=0.801
Bilirubin (µmol/L)		22.6±1.96	25.5±3.59	p=0.427
Prothrombin time (s)		14.5±0.31	14.3±0.33	p=0.565
ALBI score		−2.35±0.05	−2.46±0.07	p=0.863
Child-Pugh stage	A	108 (65%)	70 (61%)	p=0.732
	B	41 (25%)	32 (28%)	
	C	16 (10%)	13 (11%)	
BCLC stage	0+A	44 (23%)	23 (19%)	p=0.756
	B	16 (8%)	9 (7%)	
	C	89 (47%)	62 (52%)	
	D	41 (22%)	26 (22%)	
TNM stage	I	83 (44%)	43 (36%)	p=0.192
	II	31 (16%)	27 (22%)	
	IIIA+IIIB	51 (27%)	35 (29%)	
	IV	25 (13%)	15 (13%)	
Spontaneous tumour haemorrhage		1 (1%)	5 (4%)	p=0.023*
First-line treatment received	OLTx	5 (3%)	1 (1%)	p=0.708
	Resection	3 (2%)	3 (2%)	
	Ablation	21 (11%)	18 (15%)	
	TACE	41 (21%)	19 (16%)	
	SIRT	11 (6%)	8 (7%)	
	Medical	10 (5%)	7 (6%)	
	Supportive care	99 (52%)	64 (53%)	
In surveillance programme		75 (39%)	45 (38%)	p=0.727
Surveillance adherence over previous year	Consistent	56 (78%)	21 (48%)	p=0.004**
	Inconsistent	8 (11%)	13 (29%)	
	Missed	8 (11%)	10 (23%)	
Mode of incident surveillance test	US	48 (75%)	22 (69%)	p=0.218
	AFP alone	15 (23%)	8 (25%)	
	CT/MRI	1 (2%)	2 (6%)	
Type of incidental finding	Primary care—routine	19 (24%)	11 (27%)	p=0.826
	Secondary care—routine	13 (16%)	9 (22%)	
	Primary care—acute	4 (5%)	1 (3%)	
	Secondary care—acute	44 (55%)	19 (48%)	

Continuous data are shown as mean±SE of mean, with median in parentheses. Categorical datasets have been analysed by Pearson’s χ^2^ tests, with p values for continuous data determined by t-test or Mann-Whitney U tests.

*p<0.05; **p<0.005.

AFP, alpha-fetoprotein; AIH, autoimmune hepatitis; ALBI, albumin-bilirubin; ARLD, alcohol-related liver disease; BCLC, Barcelona Clinic Liver Cancer; CLD, chronic liver disease; HBV, hepatitis B virus; HCC, hepatocellular carcinoma; HCV, hepatitis C virus; HH, hereditary haemochromatosis; NAFLD, non-alcoholic fatty liver disease; OLTx, orthotopic liver transplantation; PBC, primary biliary cholangitis; SIRT, selective internal radiation therapy; TACE, transarterial chemoembolisation; TNM, tumour-node-metastases; US, ultrasound.

In the two HCC cohorts, 120 patients with known cirrhosis were undergoing formal surveillance prior to detection of their cancer, including 75 in the prepandemic cohort and 45 in pandemic cohort. Of these, 85% had their HCC detected by surveillance in the prepandemic cohort versus only 71% in the pandemic cohort (HCC detected by surveillance/incidentally/symptomatically; 64/7/4 prepandemic vs 32/3/10 in the pandemic, Pearson’s χ^2^ test p=0.020). Thus, a greater proportion presented symptomatically in the pandemic year. Data on the surveillance test preceding the incident one were available in 116/120. A greater proportion in the pandemic year had inconsistent or missed surveillance compared with the prepandemic year (consistent/inconsistent/missed prepandemic vs pandemic; 56/8/8 vs 21/13/10, Pearson’s χ^2^ test p=0.004) (table 1). In keeping with this, the interval between the incident investigation and previous surveillance test was longer in the pandemic year, although not significantly so (median 8.1 vs 6.2 months, p=0.150). Notably, of those cases where the HCC was detected by surveillance in the pandemic year, the size of the incident lesion was not significantly different (median 30 mm vs 24 mm, p=0.625). However, of the 32 cases detected via surveillance in the pandemic year, 12 (38%) had their incident surveillance test prior to the start of the pandemic. The number of new cases with an incident lesion first reported on a surveillance scan in the pandemic year was only 20.

The number of newly diagnosed ICCs also fell in the pandemic year, although less markedly than for HCC (prepandemic vs pandemic years: 76 vs 64, 16% reduction) (figure 1D), with symptomatic presentations remaining the most frequent (figure 1D and table 2).

**Table 2:** Clinical details of patients with ICC

		Year		P value
		Prepandemic (March 2019–February 2020)	Pandemic (March 2020–February 2021)	
Cases diagnosed		76	64	
Mode of presentation	Surveillance	6 (8%)	1 (2%)	p=0.130
	Incidental	12 (16%)	13 (20%)	
	Symptomatic	58 (76%)	50 (78%)	
TNM stage	I	2 (3%)	0 (0%)	p=0.002**
	II	19 (25%)	3 (5%)	
	III	12 (16%)	8 (12%)	
	IV	42 (56%)	53 (83%)	
Age in years (median)		68.69±1.33 (71)	70.22±1.30 (72)	p=0.518
Gender	Male	46 (61%)	42 (66%)	p=0.534
	Female	30 (39%)	22 (33%)	
Performance status	0	19 (25%)	13 (20%)	p=0.659
	1	31 (41%)	31 (48%)	
	2	19 (25%)	12 (19%)	
	3	7 (9%)	7 (11%)	
	4	0 (0%)	1 (2%)	
Tumour diameter in mm (median)		57.54±5.72 (50)	51.34±4.34 (42)	p=0.755
First-line treatment received	OLTx	1 (1%)	0 (0%)	p=0.261
	Resection	14 (19%)	10 (16%)	
	Ablation	1 (1%)	0 (0%)	
	TACE	0 (0%)	2 (3%)	
	SIRT	3 (4%)	0 (0%)	
	Medical	20 (26%)	14 (23%)	
	Supportive Care	37 (49%)	36 (58%)	

Continuous data are shown as mean±SE of mean, with median in parentheses. Categorical datasets have been analysed by Pearson’s χ^2^ tests, with p values for continuous data determined by t-test or Mann-Whitney U tests.

*p<0.05; **p<0.005.

ICC, intrahepatic cholangiocarcinoma; OLTx, orthotopic liver transplantation; SIRT, selective internal radiation therapy; TACE, transarterial chemoembolisation; TNM, tumour-node-metastases.

### HCCs were larger and ICCs more advanced in the pandemic year

For patients with HCC, there were no significant differences in the Barcelona Clinic for Liver Cancer (BCLC) (figure 1E and table 1) or tumour-node-metastases (TNM) stages (table 1). However, HCCs diagnosed during the pandemic year were significantly larger (figure 1F and table 1) (60±4.6 mm vs 48±2.6 mm, p=0.017) and were more often complicated by spontaneous haemorrhage (5/120 compared with 1/190, Pearson’s χ^2^ p=0.023) (table 1).

For ICC cases, the difference in tumour size was not significantly different (figure 1G and table 2) (median 50 mm vs 42 mm, p=0.755). However, a greater proportion were staged as TNM stage IV, with fewer as TNM stage I or II (% TNM I/II/III/IV prepandemic vs pandemic year; 3/25/16/56 vs 0/5/12/83, Pearson’s χ^2^ p=0.002) (figure 1H and table 2).

### Treatments were available and appropriate for stage, with reduced waiting times for interventional procedures

The majority of patients with HCC in both prepandemic and pandemic years received supportive care (52% and 53%, respectively). Although the numbers treated were fewer, there were no significant differences in the types of treatments offered to those presenting to us (table 1). There were features of note, despite lack of significance. Only one patient with newly diagnosed HCC received a liver transplant in our centre in the pandemic year, compared with five presenting the year before. This possibly reflected the impact COVID-19 had on numbers of patients presenting to us, as well as changes in the transplant allocation system, as outlined elsewhere.^13–15^ There was a lesser impact on resection and ablation, as elective cancer surgeries continued in our centre. Notably, the numbers of patients receiving first-line transarterial chemoembolisation (TACE) halved, from 41 to 19, again reflecting fewer patients but also—despite the impact of COVID-19 and offering hope for those presenting now—two new therapies for advanced HCC being approved by the National Institute for Health and Care Excellence (NICE) during the pandemic. These include selective internal radiotherapy treatment (SIRT),^16^ which facilitated the treatment of patients with arterial therapies despite increased tumour size. Although numbers were small, in contrast to the fall in TACE treatments, SIRT treatments increased as a percentage. Standard medical therapy with the oral tyrosine kinase sorafenib could be supervised remotely and was still used. In addition though, treatment with the combination atezolizumab and bevacizumab was also approved by NICE,^17^ having been demonstrated to be superior to sorafenib in all clinical endpoints in 2020.^18^ This combination was also offered to our patients, towards the end of 2020.

Data on the time from MDT discussion to first treatment were available for the majority of patients with HCC undergoing active treatment (116/147; 79%) and was significantly shorter in the pandemic year compared with the prepandemic year (median 1.6 vs 2.3 months, p=0.001). This reflected shorter waits with fewer patients, as well as patients with cancer being prioritised for radiology or surgical therapies over patients without cancer.

Treatments administered to patients with ICC were also similar in the pandemic and prepandemic years, with 19% versus 16% receiving surgery, 26% versus 23% medical therapies and 49% vs 58% supportive care (table 2). The time from MDT discussion to first treatment was available for 46/65 (71%) of patients with ICC undergoing active treatment, with no significant difference in treatment times (prepandemic vs pandemic; median 1.6 vs 1.4 months, p=0.644).

### Impact on survival

For patients with HCC, univariate survival was similar for those presenting in the pandemic and prepandemic years, although follow-up is ongoing (online supplemental figure 1A). In the combined cohorts and individually, median survival was—as expected—highly significantly associated with BCLC stage (online supplemental figure 1B), as well as being significantly better for those detected by surveillance, compared with those detected incidentally or presenting symptomatically (online supplemental figure 1C). Patients in formal surveillance programmes had better survival compared with those who were not (29.3 vs 13.9 months, log-rank p<0.001) (online supplemental figure 1D), with patients undergoing consistent surveillance having better survival compared with those with inconsistent or missed surveillance (online supplemental figure 1E). The numbers are small and lead time bias unaccounted for, but the data were in keeping with previous reports.^19^

10.1136/bmjgast-2021-000794.supp1 Supplementary data

For patients with ICC, the median survival was significantly worse during the pandemic year compared with the prepandemic year (4.7 vs 11.4 months, log-rank p=0.028) (online supplemental figure 1F), attributed to more cases presenting with advanced TNM stage (online supplemental figure G, H).

### Outcomes of patients with HCC after COVID-19 infection

Nine per cent (11/120) of patients diagnosed with HCC in the pandemic year acquired COVID-19 infection, as documented in their tertiary centre record. None died directly from COVID-19 infection. Of the six who acquired COVID-19 before or synchronous to their HCC diagnosis, two went on to receive active treatment for their HCC and four supportive care. Of the five who acquired COVID-19 following their HCC diagnosis, three died of advanced HCC, one continued to receive active treatment and one supportive care (online supplemental figure 2).

10.1136/bmjgast-2021-000794.supp2 Supplementary data

## Discussion

This study investigated the impact of the COVID-19 pandemic on patients with PLC in our region in the first year of the pandemic. We report a 37% decrease in the number of new HCCs diagnosed during the pandemic year—a marked contrast to the steady increases in previous years and suggesting incident cases went undiagnosed during this period. Similar reductions have been reported in the west of Scotland as well as in a large multicentre Asian study.^20 21^

In the pandemic year, symptomatic presentation for HCC moved from being the least common mode of presentation to the the most common—accounting for 40% of all cases. The numbers detected by surveillance or incidentally were halved. ‘Incidental’ patients were those referred from primary care after routine checks for age or chronic conditions or those referred from secondary care after investigation for symptoms unrelated to their cancer. The impact on lost appointments or routine reviews was not possible to quantify. However, of our patients with cirrhosis in surveillance programmes, we have been able to analyse the data for those who have already had their cancer detected. In the prepandemic year, surveillance was suboptimal in a fifth of patients, with 5% presenting symptomatically. This was disappointing, but in the pandemic year, surveillance was even worse, with 52% of patients having suboptimal surveillance and 22% presented symptomatically. On a more encouraging note, for those patients who remained well and who did attend for their delayed surveillance scans, with a cancer subsequently detected, the median size of their incident lesions was not significantly greater in the pandemic year. Survival data collection is ongoing, but for the patients in our care, we expect it to be similar. Although there were fewer HCC cases in the pandemic year, the fall in earlier TNM and BCLC stages was not significantly different. This reflects the prepandemic reality in our region, plagued by socioeconomic deprivation, high levels of obesity and alcohol excess, where the majority of patients typically present with advanced disease.^9^ Importantly, despite the challenges posed by the pandemic and excluding liver transplantation, patients reaching us received appropriate treatment according to their disease stage. These included treatments with SIRT and combination atezolizumab and bevacizumab for advanced disease. However, as the disruption to routine care continues, we anticipate that more and more patients will miss routine monitoring or surveillance appointments and present symptomatically. It was notable that the most significant aetiological reduction was in the number of new HCV-HCC cases. Our patients with HCV-HCC are typically younger^9^ with fewer comorbidities, with those missing surveillance not yet ‘symptomatic’ nor coming to the attention-of-care services for other reasons.

Considering our patients with ICC during the pandemic year, numbers fell only slightly, with symptomatic presentations remaining the most frequent. This was not unexpected, as ICC is a tumour type not typically detected by surveillance. Those few cases that were detected by surveillance were in patients with an underlying cirrhosis. Unfortunately, 95% of the pandemic cases had stage III/IV ICC, compared with 72% previously, consequently with poorer survival.

Only 9% of the patients diagnosed with HCC contracted COVID-19, which may be attributable to the success of our government advising ‘shielding’ for patients with chronic liver disease. Emerging cumulative data does indicate that patients with chronic liver disease acquiring COVID-19 have a higher mortality risk.^22^ While COVID-19 infections and related deaths may have been higher without the measures taken, in our cohort patient deaths were consequent to advanced cancer stage. The risk of acquiring COVID-19 needs to be balanced against that of missed HCC diagnoses and lost opportunities for treatments. As awareness of guidance and numbers of adults vaccinated rises, the benefits of resuming routine care must be re-evaluated.

In line with international consensus recommendations, we advise that HCC surveillance in patients with cirrhosis is resumed as rapidly as possible, with delays limited to under 2–3 months if possible.^23^ Our data support this, as this level of delay has not detrimentally impacted the outcome of those who have engaged with healthcare and attended for their delayed surveillance appointments. In addition, we wish to highlight to healthcare funders, the need to support both routine primary and secondary care services—given it was the proportion of cancers detected incidentally in our region that fell most markedly. Primary care services have been put under considerable strain as a result of the pandemic, with ongoing reductions in patient contact and negative impacts on the management of chronic diseases, which we associate with the loss of our incidental HCC cases.^24 25^ We advise that these services need urgent reinstatement and our now vaccinated patients should be encouraged to attend. Not only is the early detection of liver cancers associated with cure,^26^ there are now life-prolonging therapies available for those presenting with more advanced disease. Patients presenting to hospitals can benefit from treatments despite the pandemic—in line with established guidelines, with some adjustments, for example, first-line locoregional therapies rather than surgery and telemedicine appointments rather than face-to-face visits.^23 27–29^ While strict temporary measures can help to control the pandemic in the short term, we want to raise awareness of their detrimental impact if continued. By informing and educating healthcare authorities, service providers and patients, we hope to avert the numbers of patients dying with advanced stage liver cancer, without the opportunity for tertiary centre-led treatments or best supportive care.

## Data Availability

All data created during this research are openly available at https://doi.org/10.25405/data.ncl.19590727.v1
