# Supplementary figures and images for: COVID-19 and liver cancer: lost patients and larger tumours
Source: BMJ Open Gastroenterol. 2022 Apr 21;9(1):e000794. doi: 10.1136/bmjgast-2021-000794 (PMC9023844; doi:10.1136/bmjgast-2021-000794)

# Supplementary Figure 1

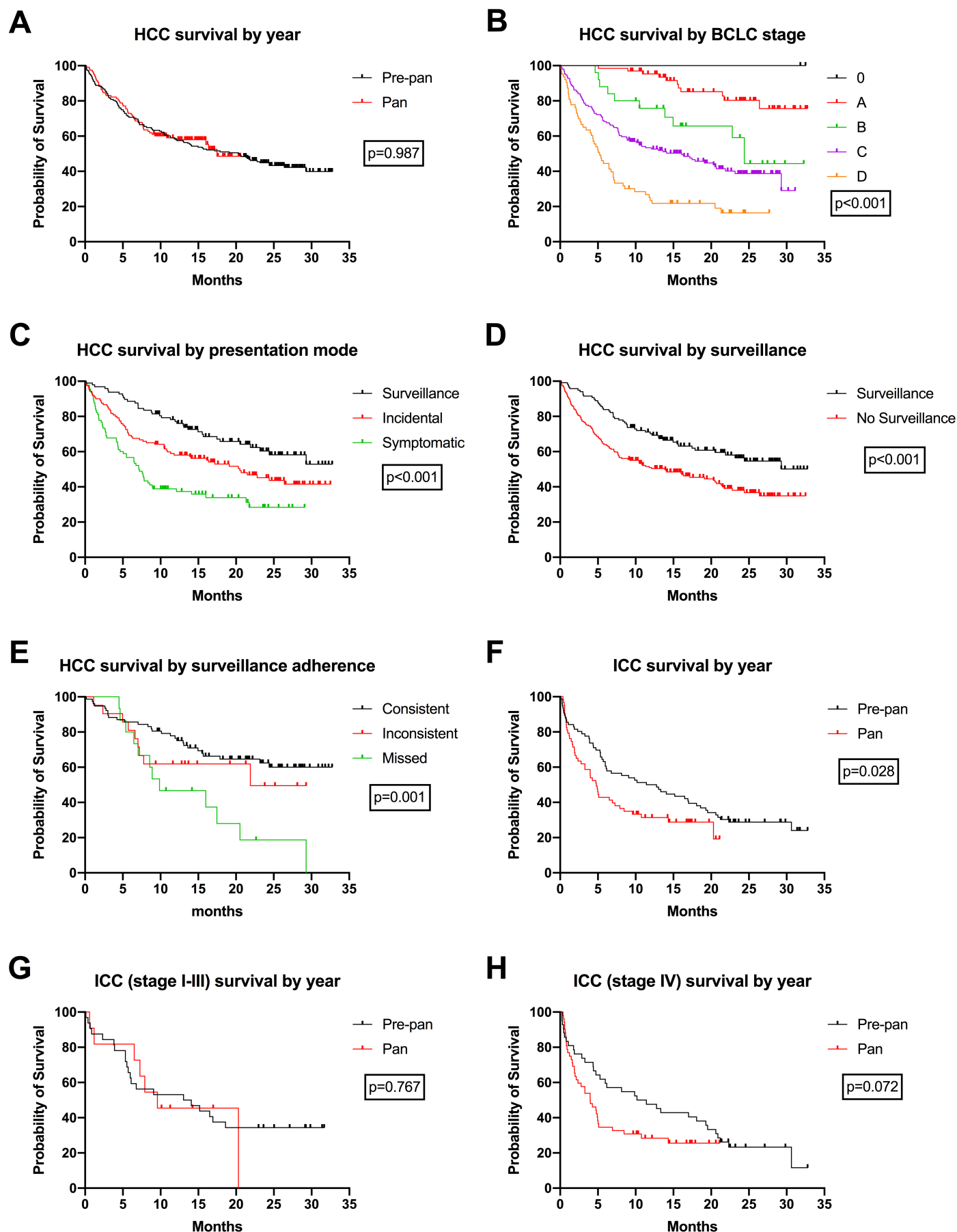

Supplement: Supplementary data [file bmjgast-2021-000794supp001.pdf]

## Supplementary Figure 2

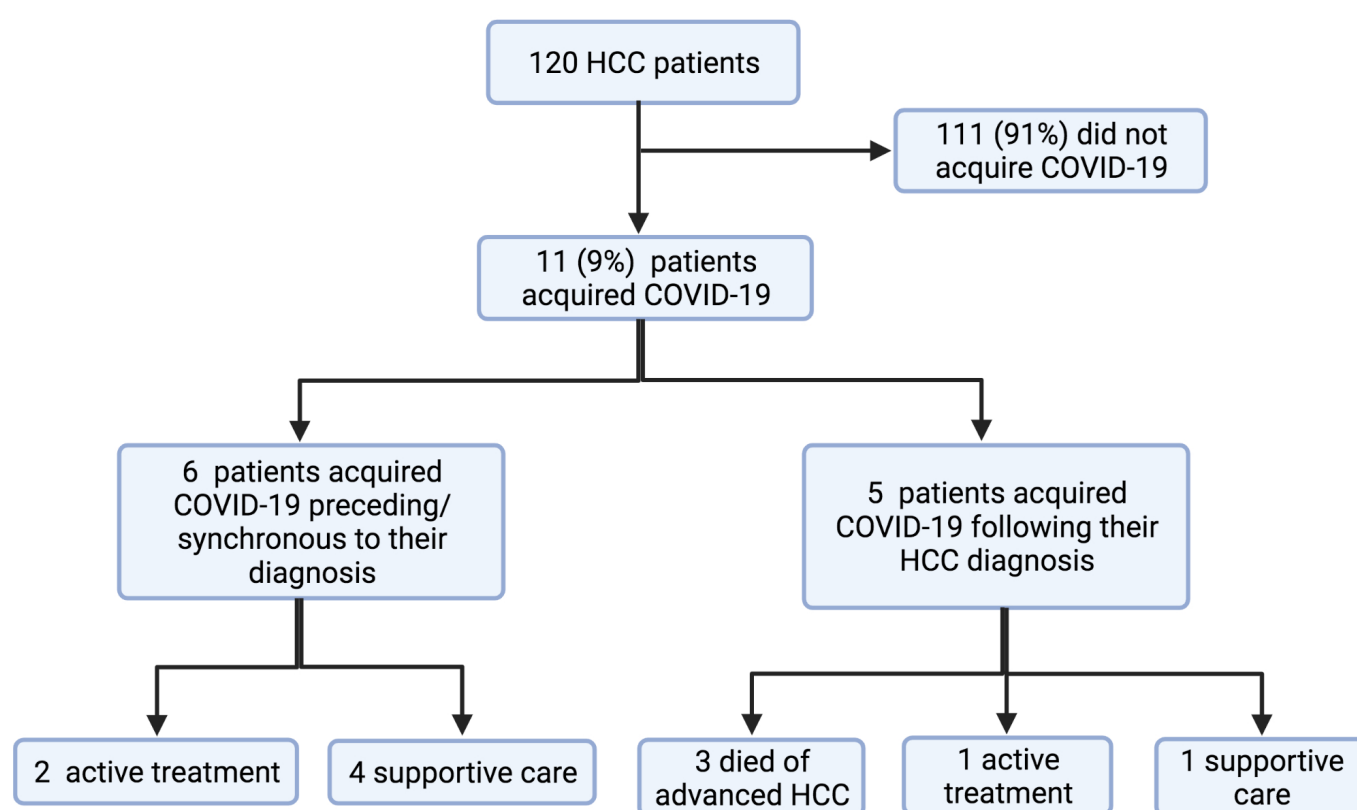

Supplement: Supplementary data [file bmjgast-2021-000794supp002.pdf]
